# Supplementary material for: Factors influencing SARS-CoV-2 IgG test sensitivity: A Bayesian analysis of seroconversion and seroreversion by time since infection, test, age and disease severity
Source: PLoS One. 2026 Feb 2;21(2):e0328144. doi: 10.1371/journal.pone.0328144 (PMC12863488; doi:10.1371/journal.pone.0328144)
Supplement: S1 Fig — (PDF) [file pone.0328144.s001.pdf]

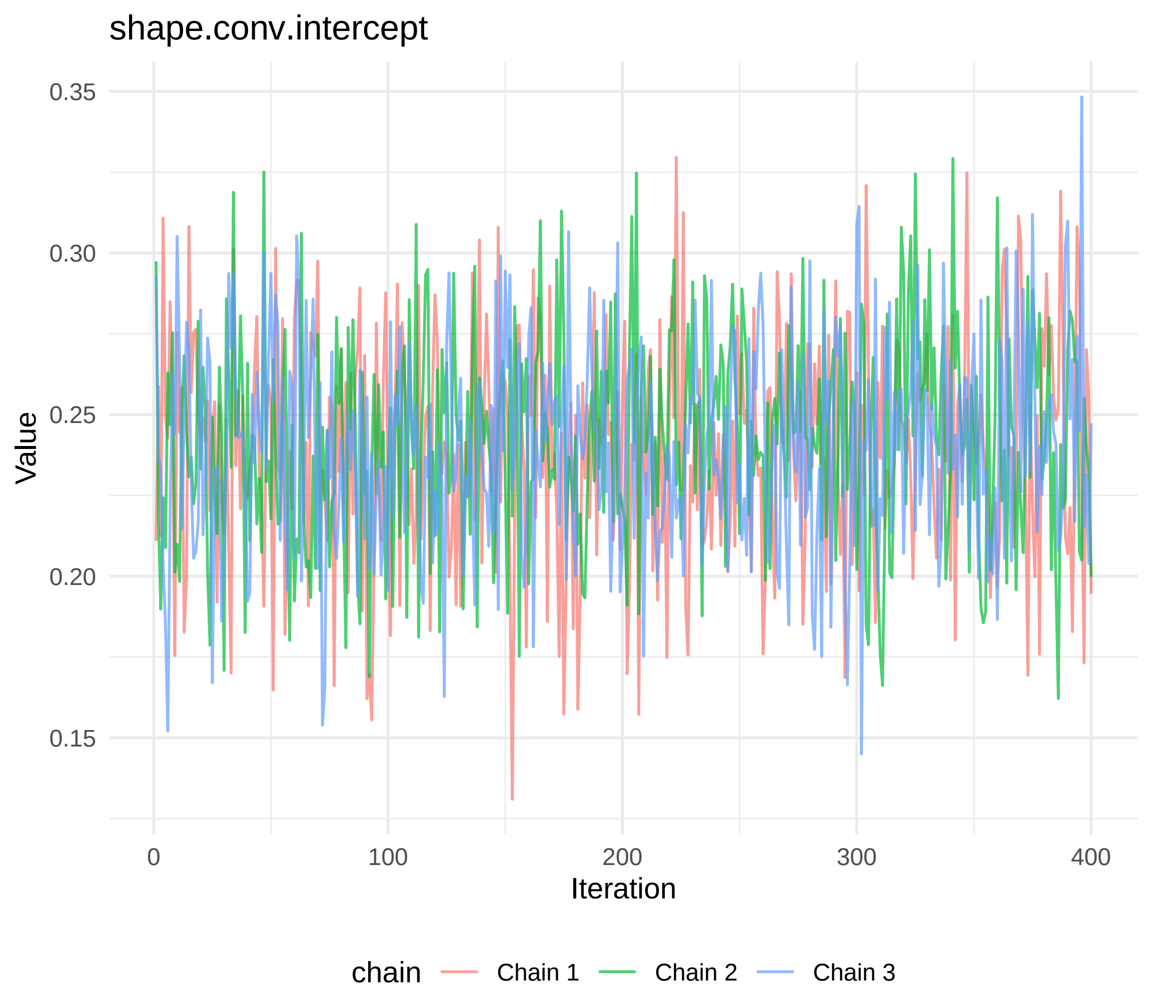
shape.conv.intercept
Value
0.35
0.30
0.25
0.20
0.15
0
100
200
300
400
Iteration
chain
Chain 1
Chain 2
Chain 3

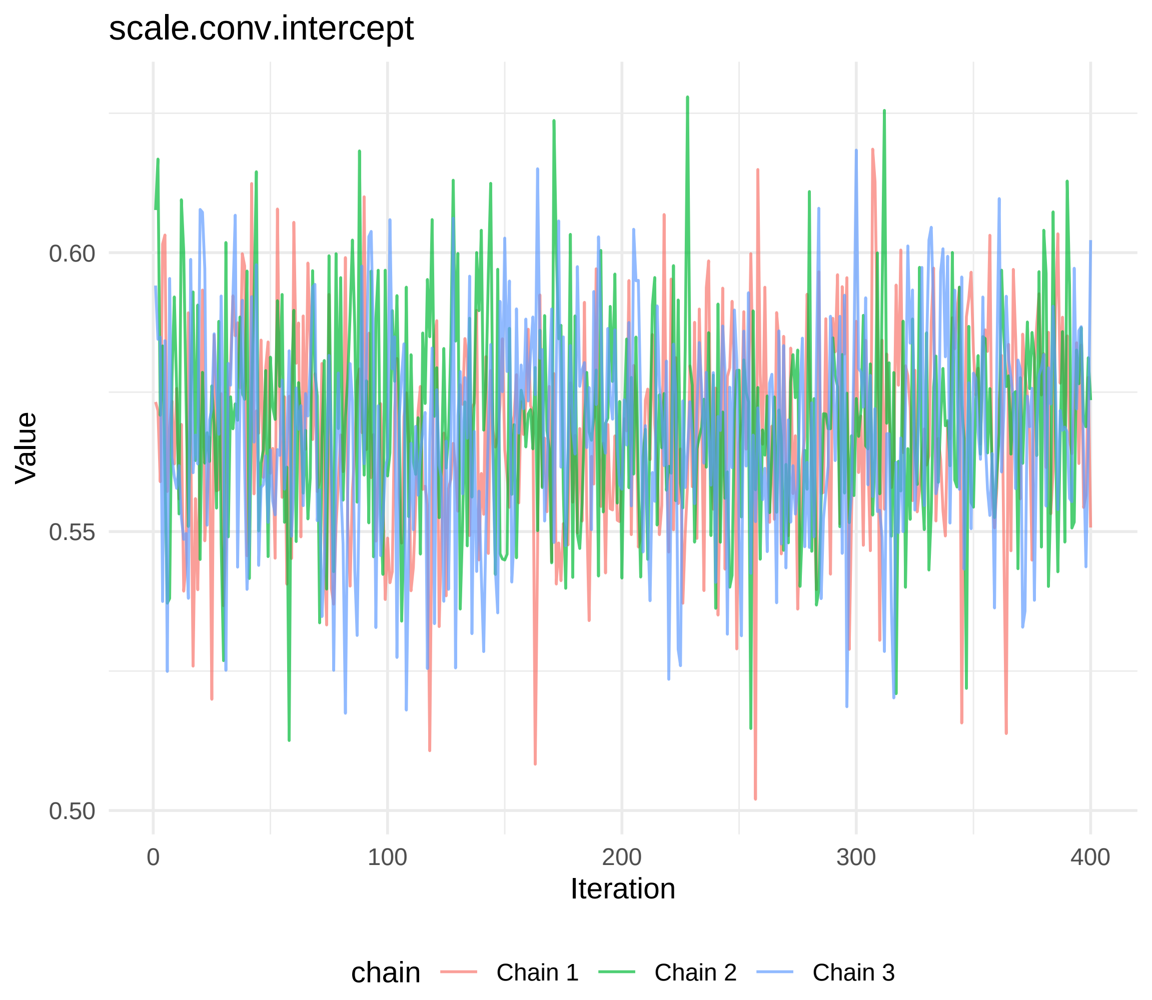
scale.conv.intercept
Value
0.60
0.55
0.50
0
100
200
300
400
Iteration
chain
Chain 1
Chain 2
Chain 3

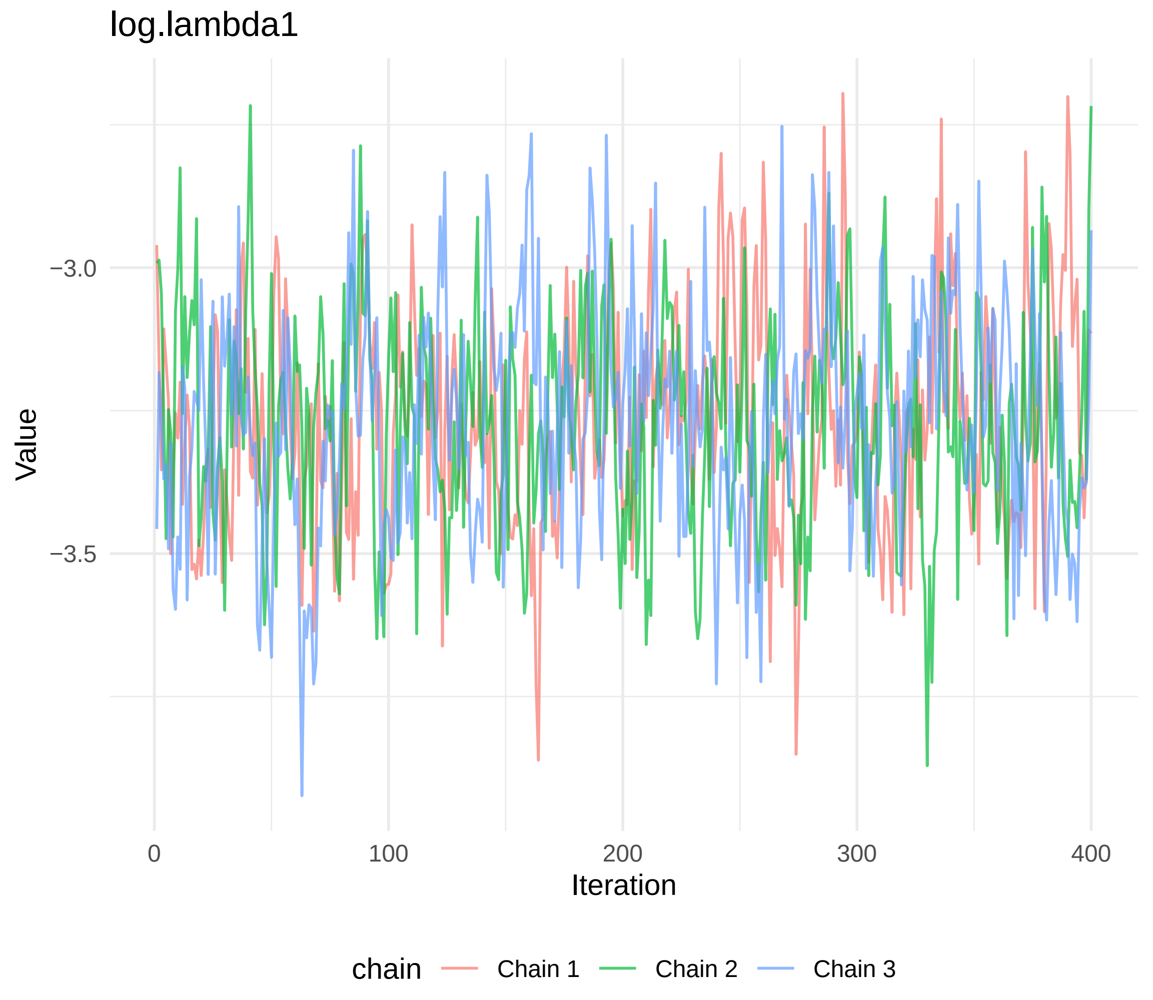
log.lambda1
Value
−3.0
−3.5
0
100
200
300
400
Iteration
chain
Chain 1
Chain 2
Chain 3

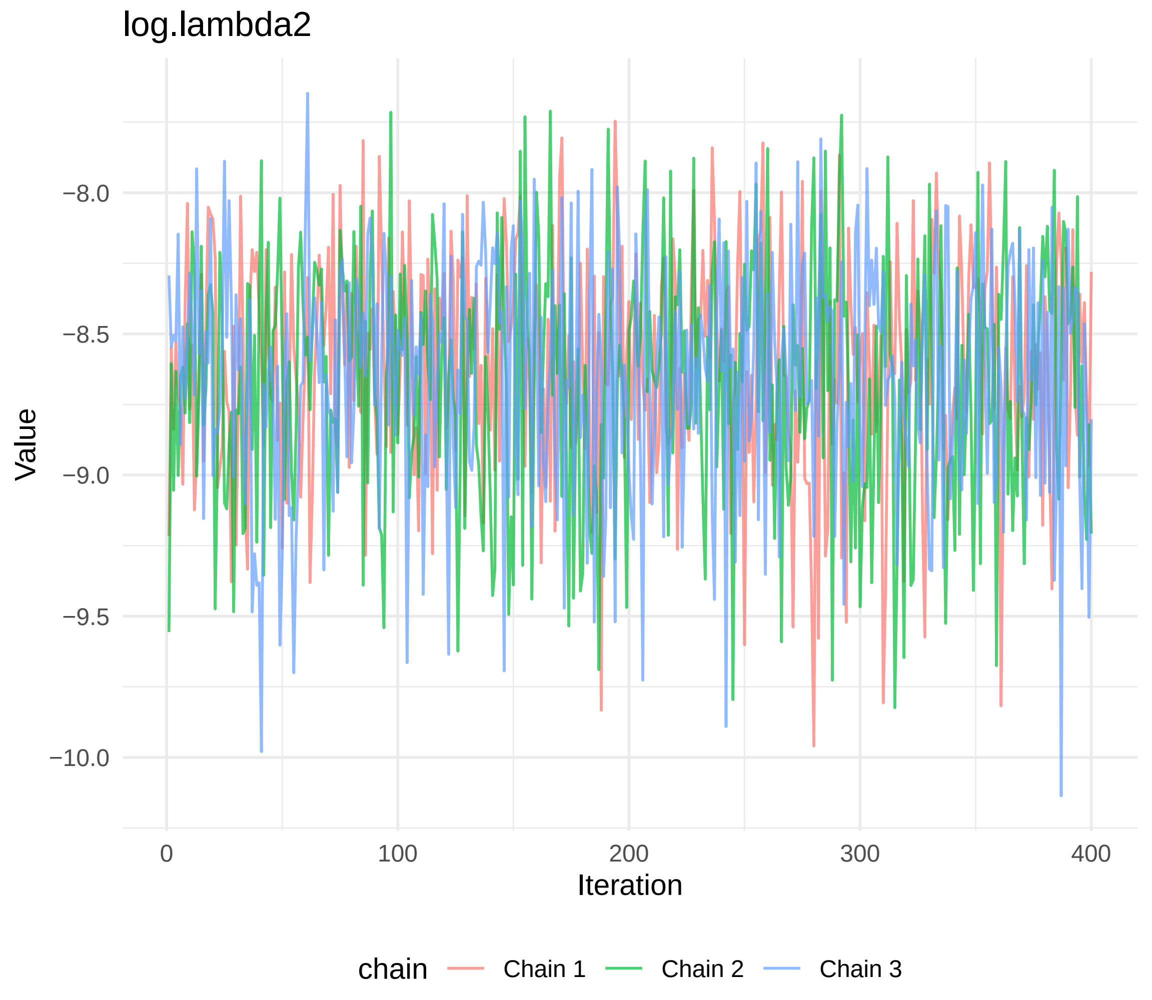
log.lambda2
Value
−8.0
−8.5
−9.0
−9.5
−10.0
0
100
200
300
400
Iteration
chain
Chain 1
Chain 2
Chain 3

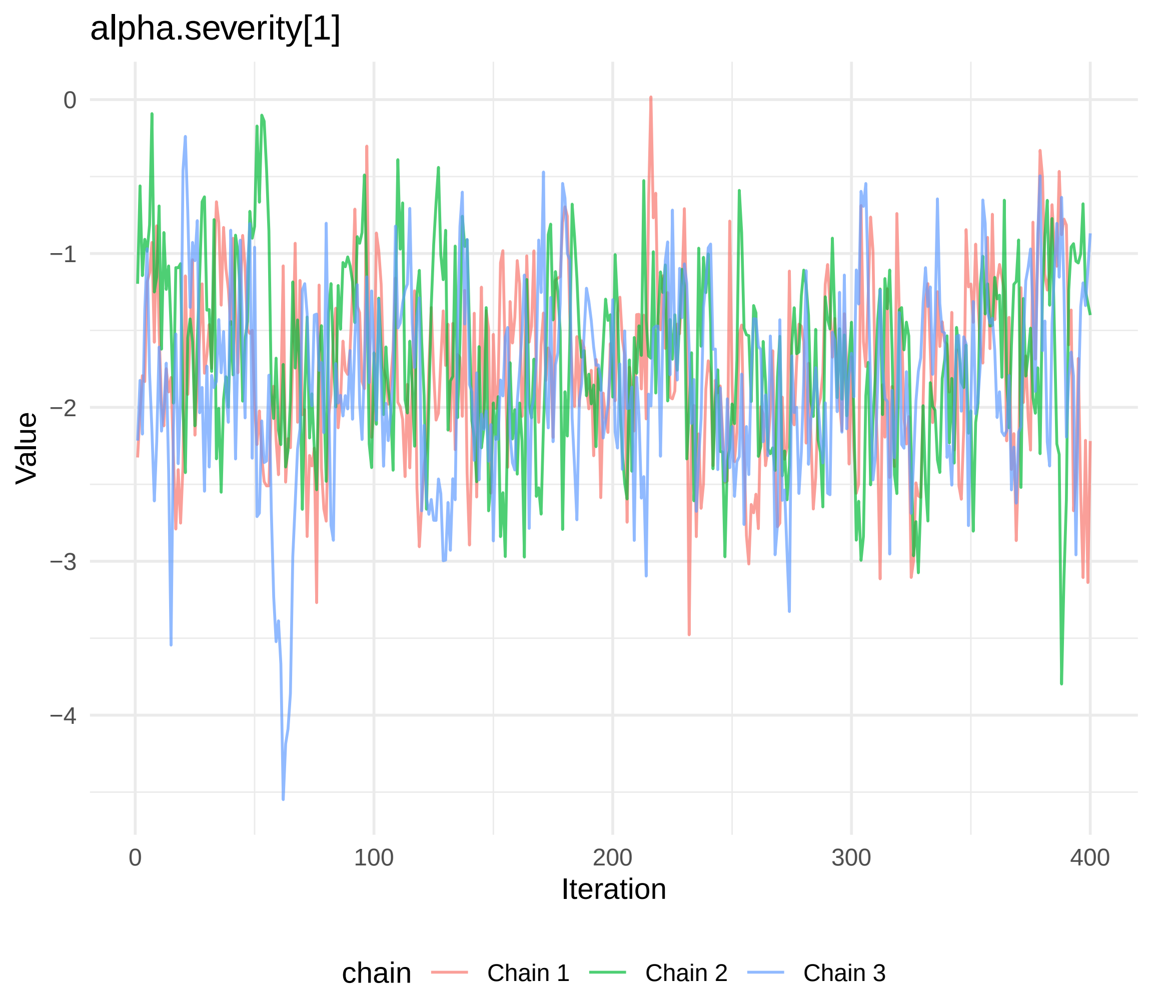
alpha.severity[1]
Value
0
−1
−2
−3
−4
0
100
200
300
400
Iteration
chain
Chain 1
Chain 2
Chain 3

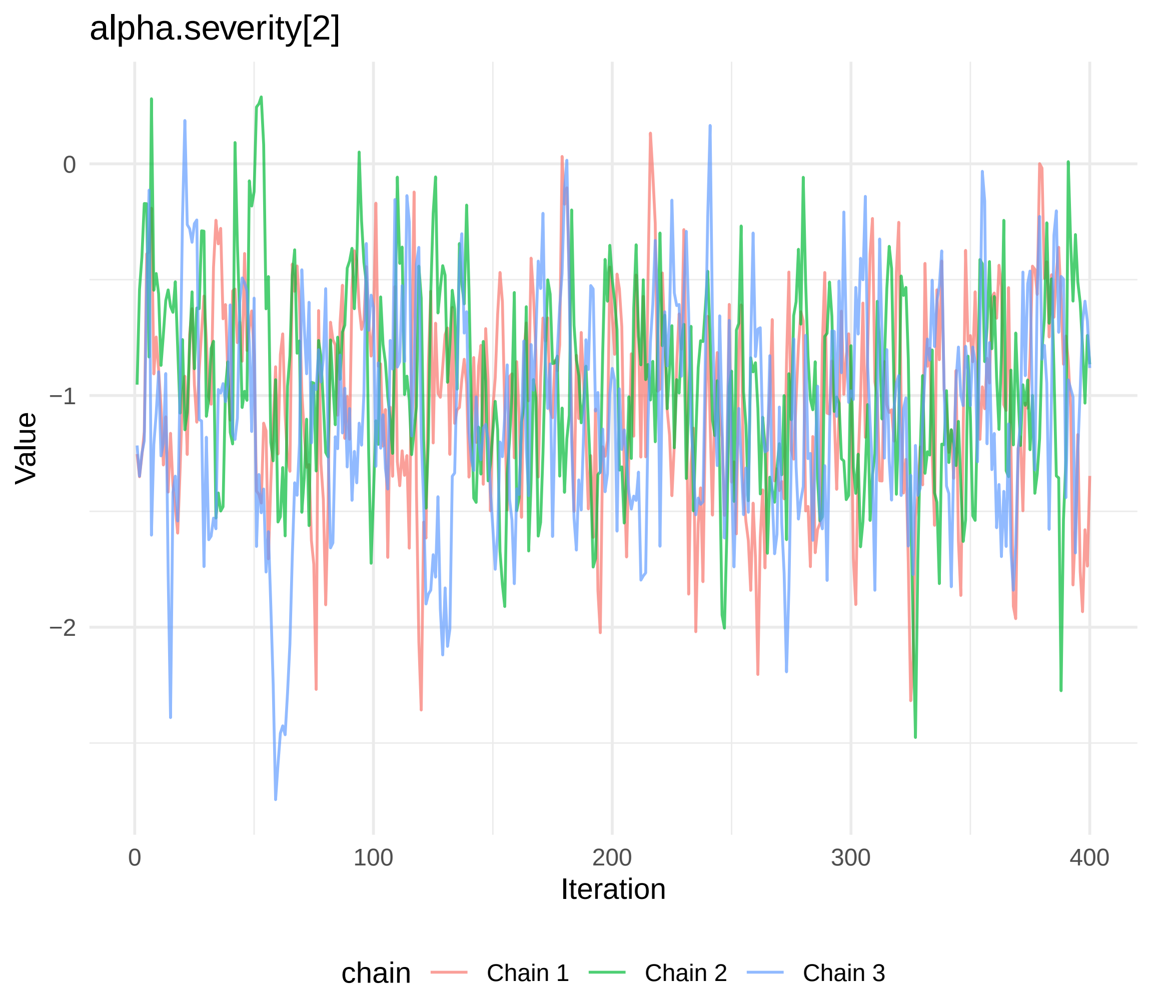
alpha.severity[2]
Value
0
−1
−2
0
100
200
300
400
Iteration
chain
Chain 1
Chain 2
Chain 3

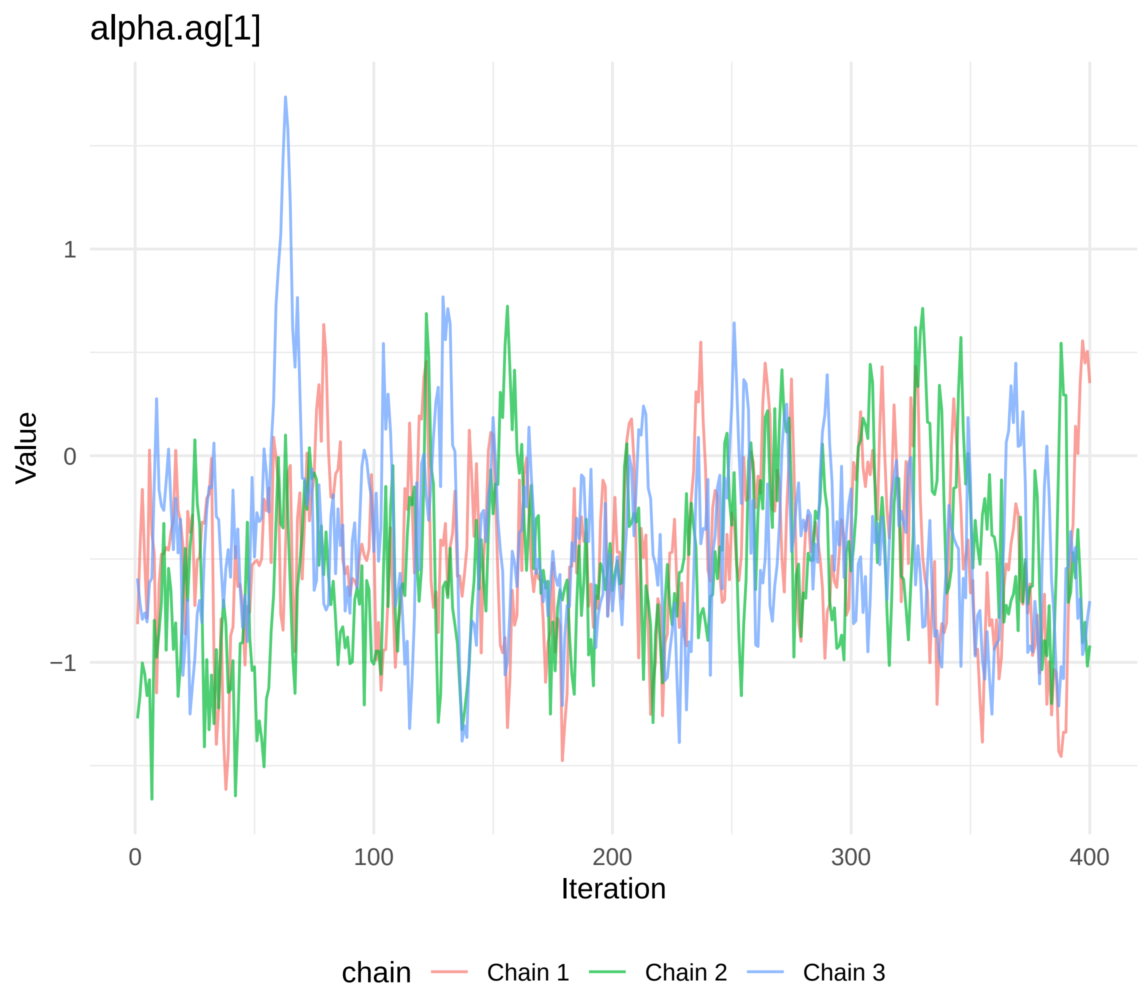
alpha.ag[1]
Value
1
0
−1
0
100
200
300
400
Iteration
chain
Chain 1
Chain 2
Chain 3

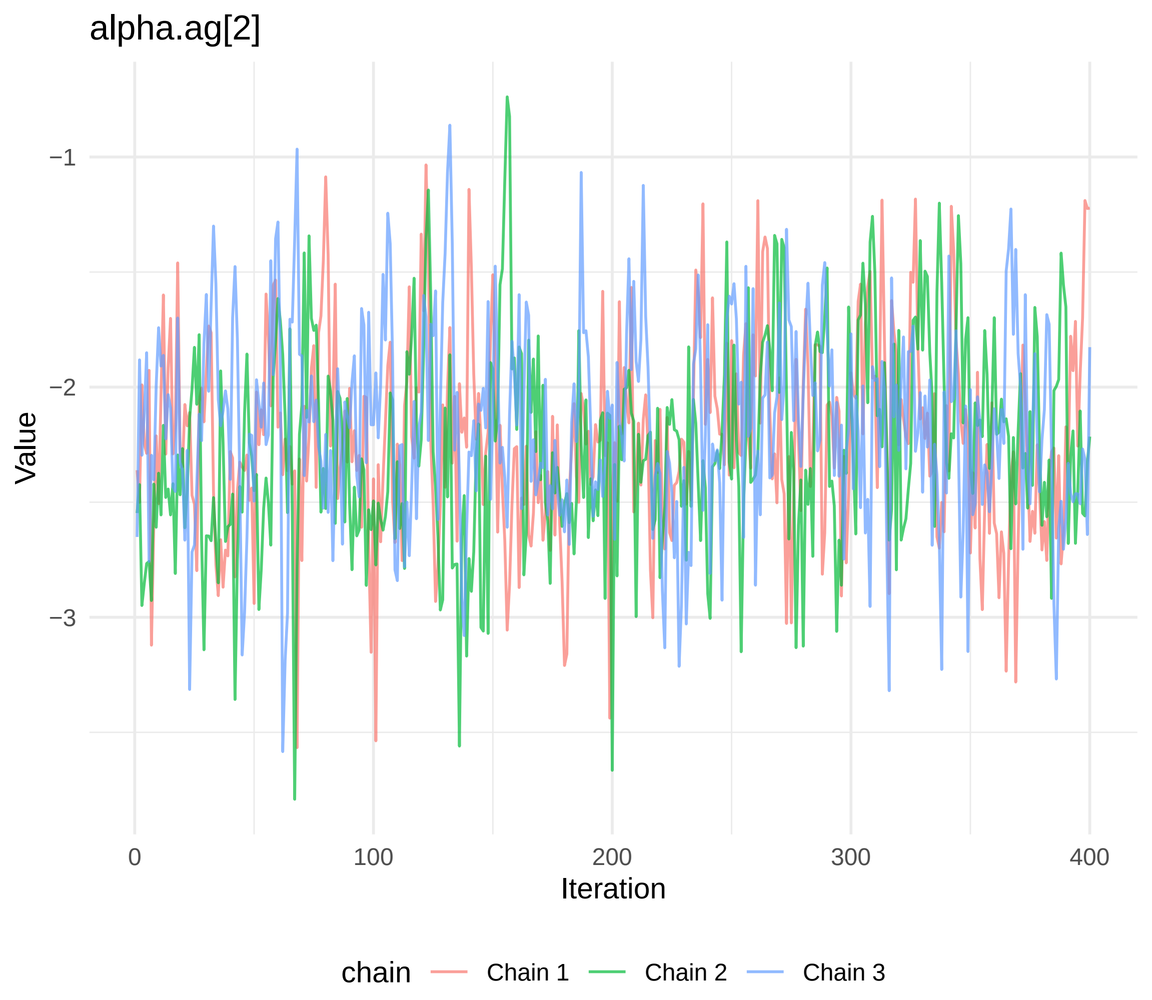
alpha.ag[2]
Value
−1
−2
−3
0
100
200
300
400
Iteration
chain
Chain 1
Chain 2
Chain 3

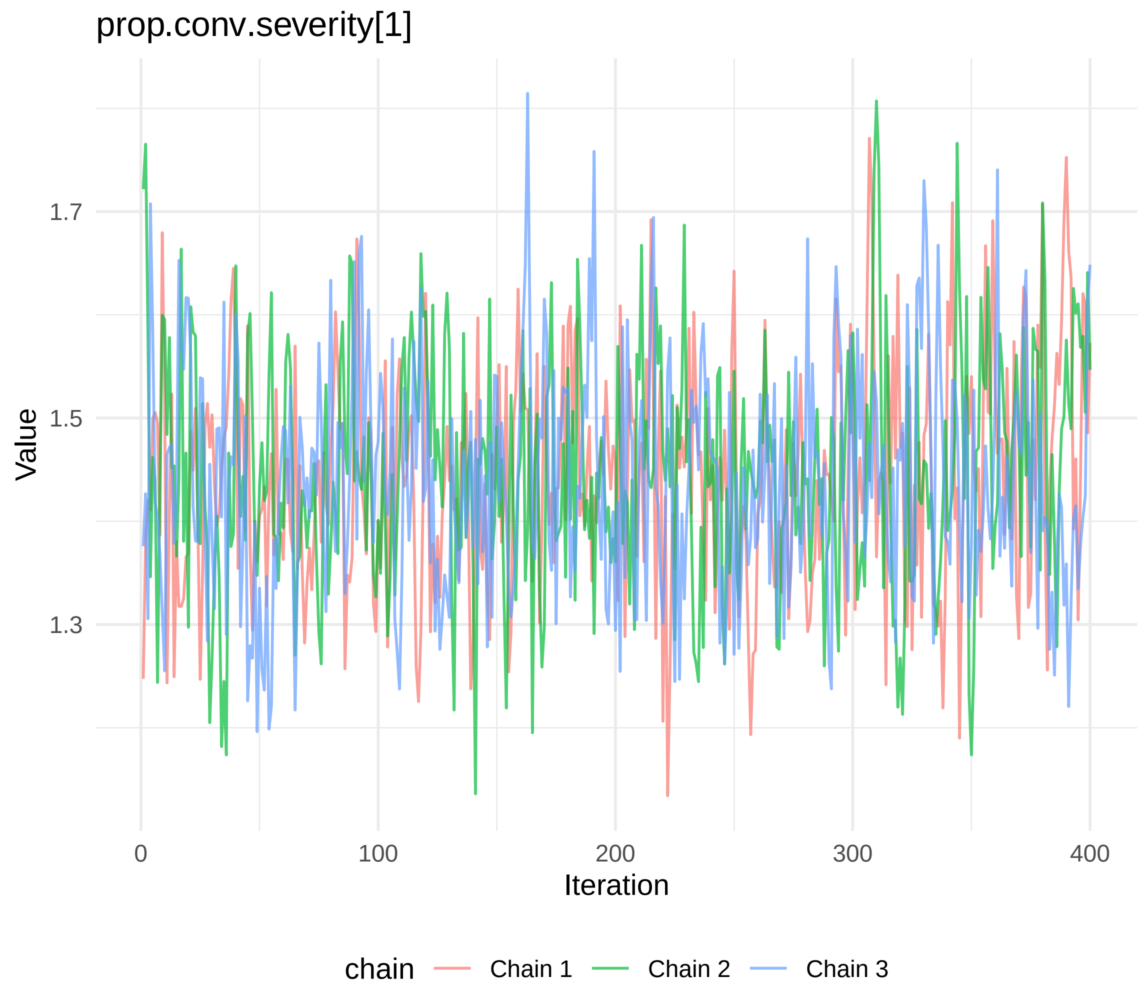
prop.conv.severity[1]
Value
1.7
1.5
1.3
0
100
200
300
400
Iteration
chain
Chain 1
Chain 2
Chain 3

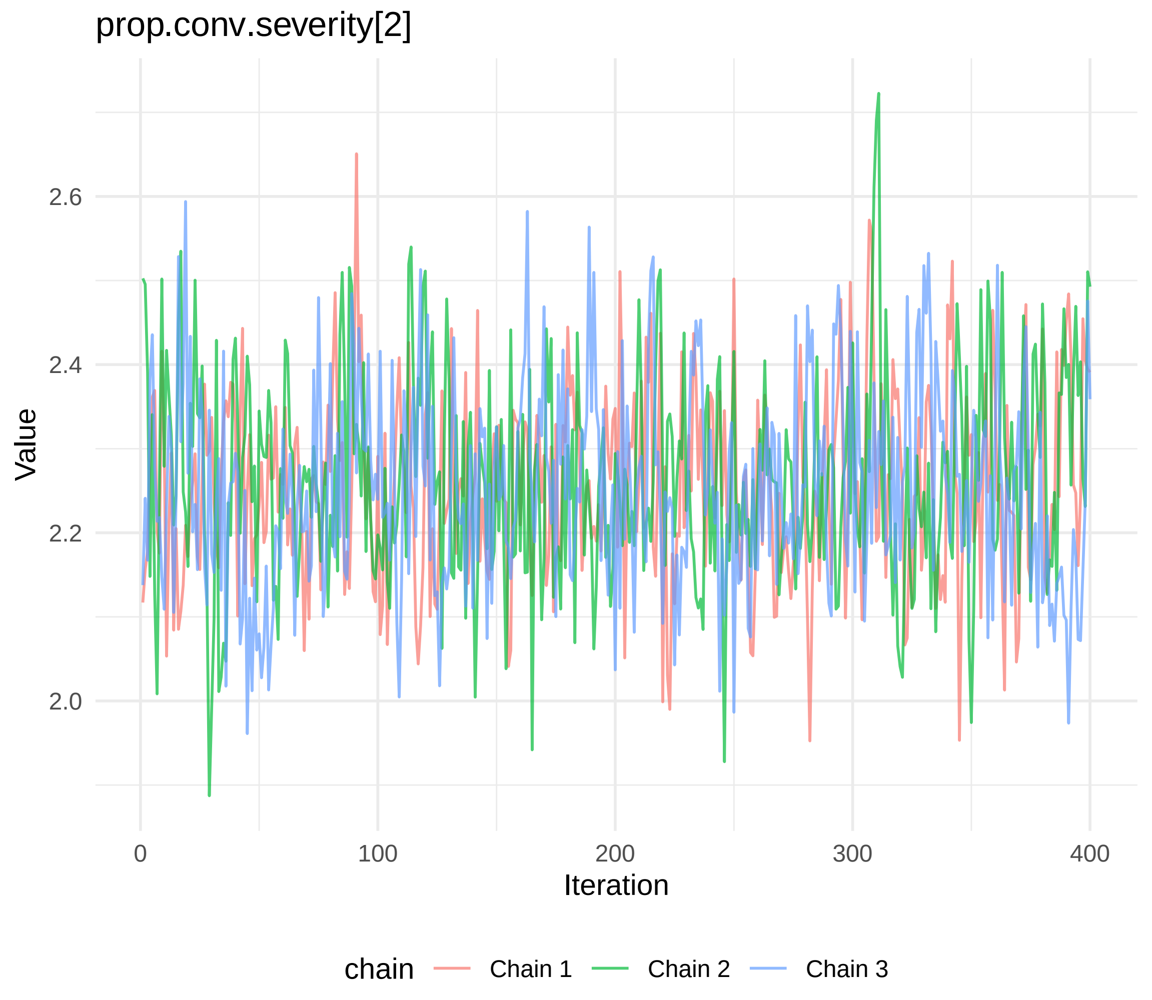
prop.conv.severity[2]
Value
2.6
2.4
2.2
2.0
0
100
200
300
400
Iteration
chain
Chain 1
Chain 2
Chain 3

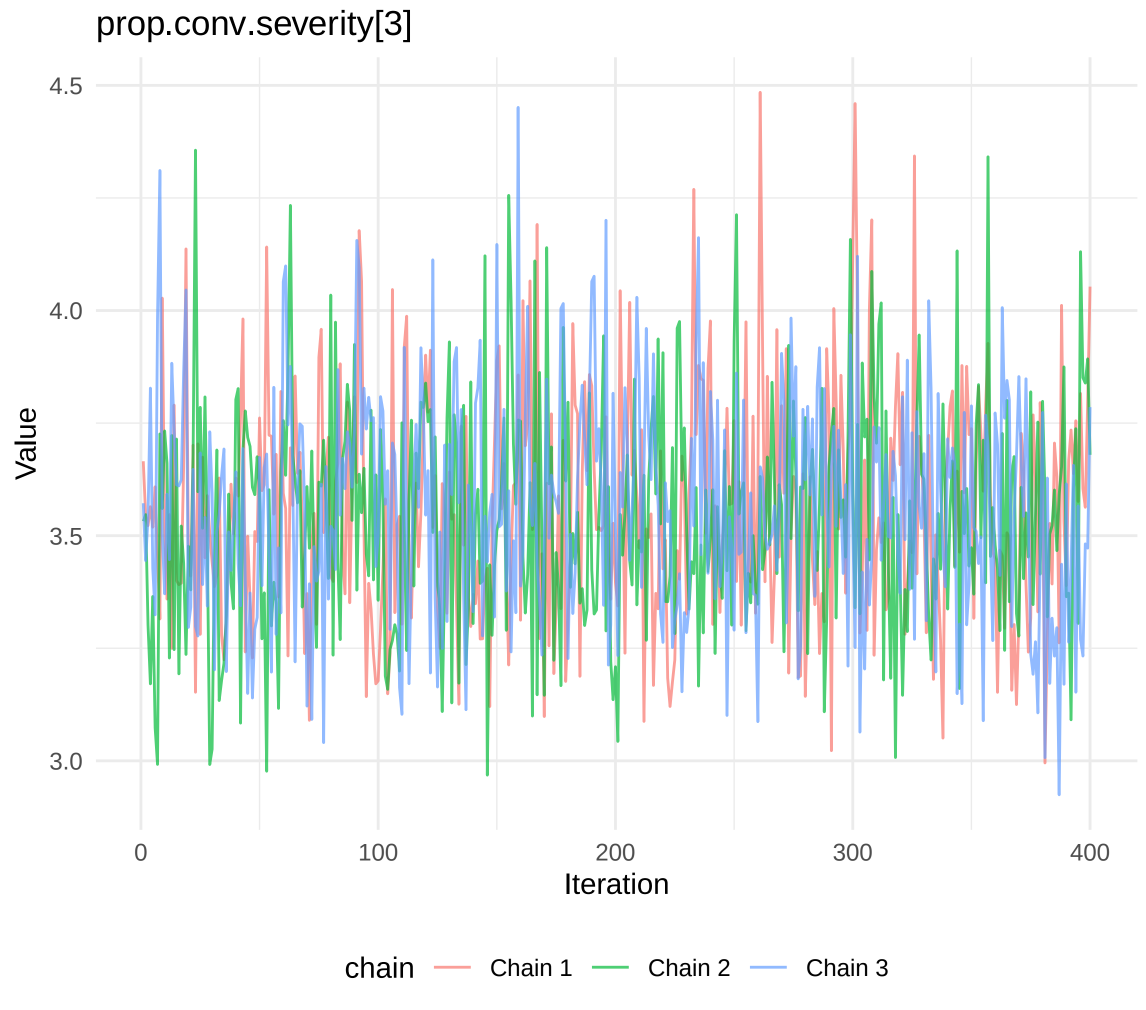
prop.conv.severity[3]
Value
4.5
4.0
3.5
3.0
0
100
200
300
400
Iteration
chain
Chain 1
Chain 2
Chain 3

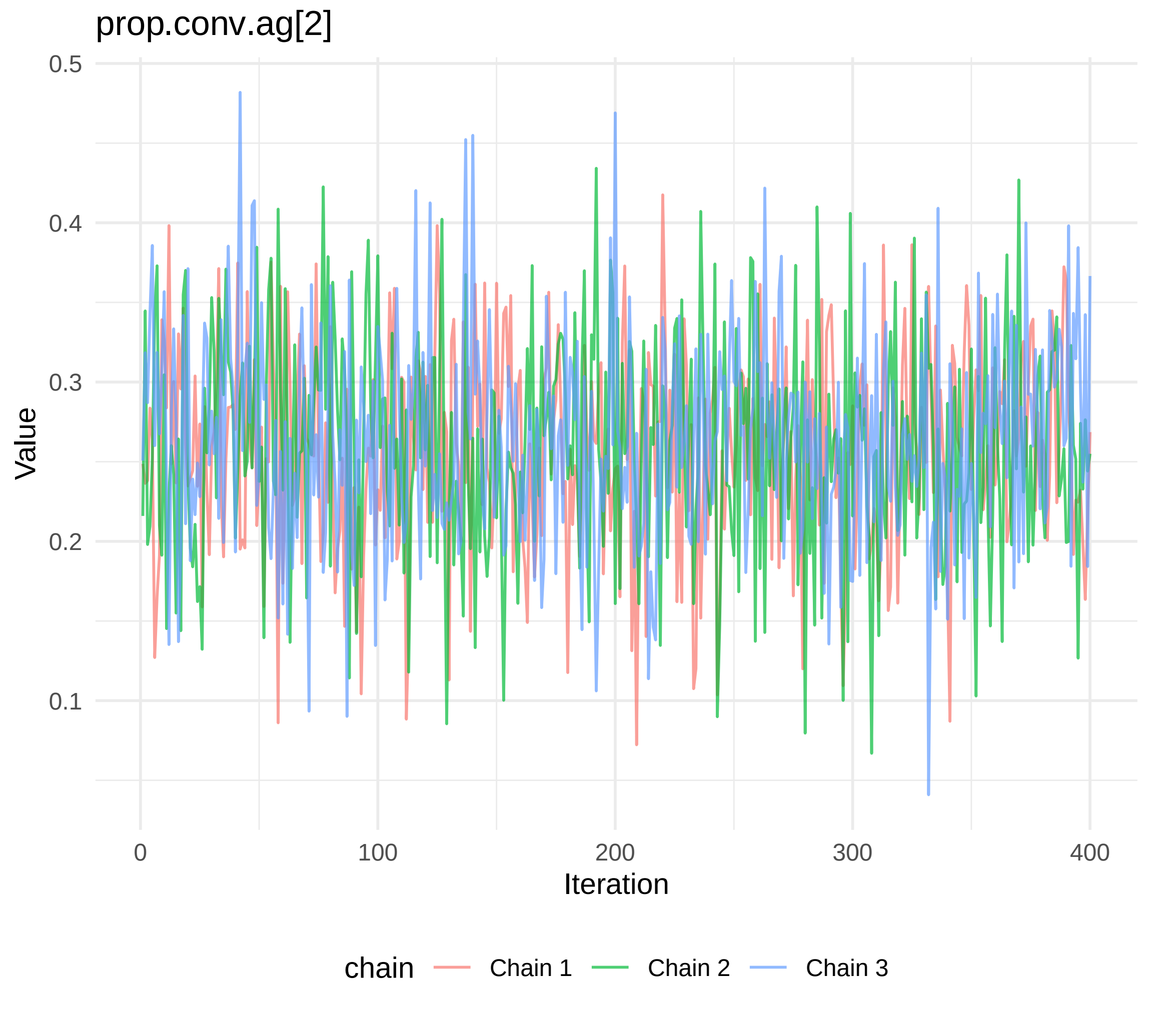
prop.conv.ag[2]
Value
0.5
0.4
0.3
0.2
0.1
0
100
200
300
400
Iteration
chain
Chain 1
Chain 2
Chain 3

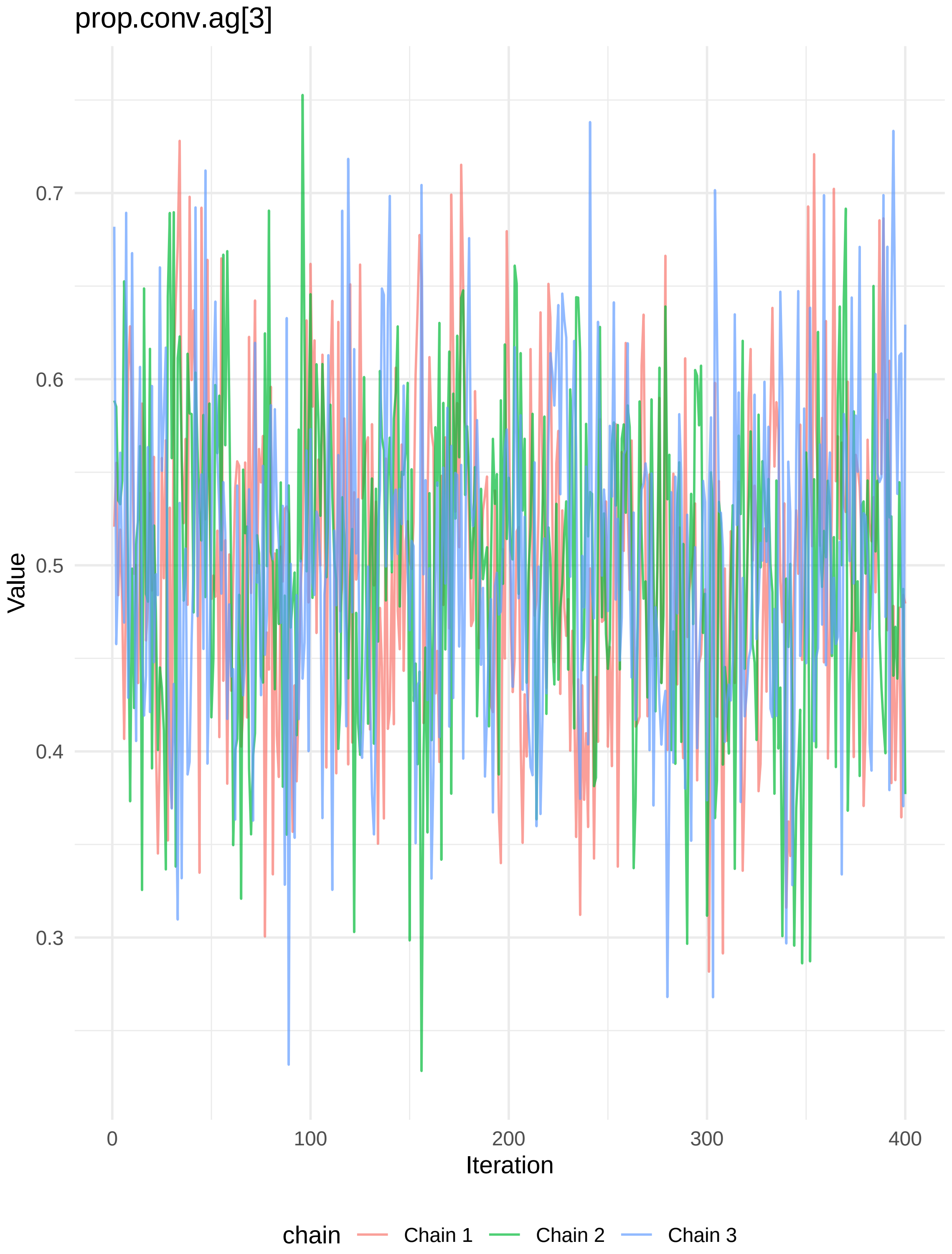

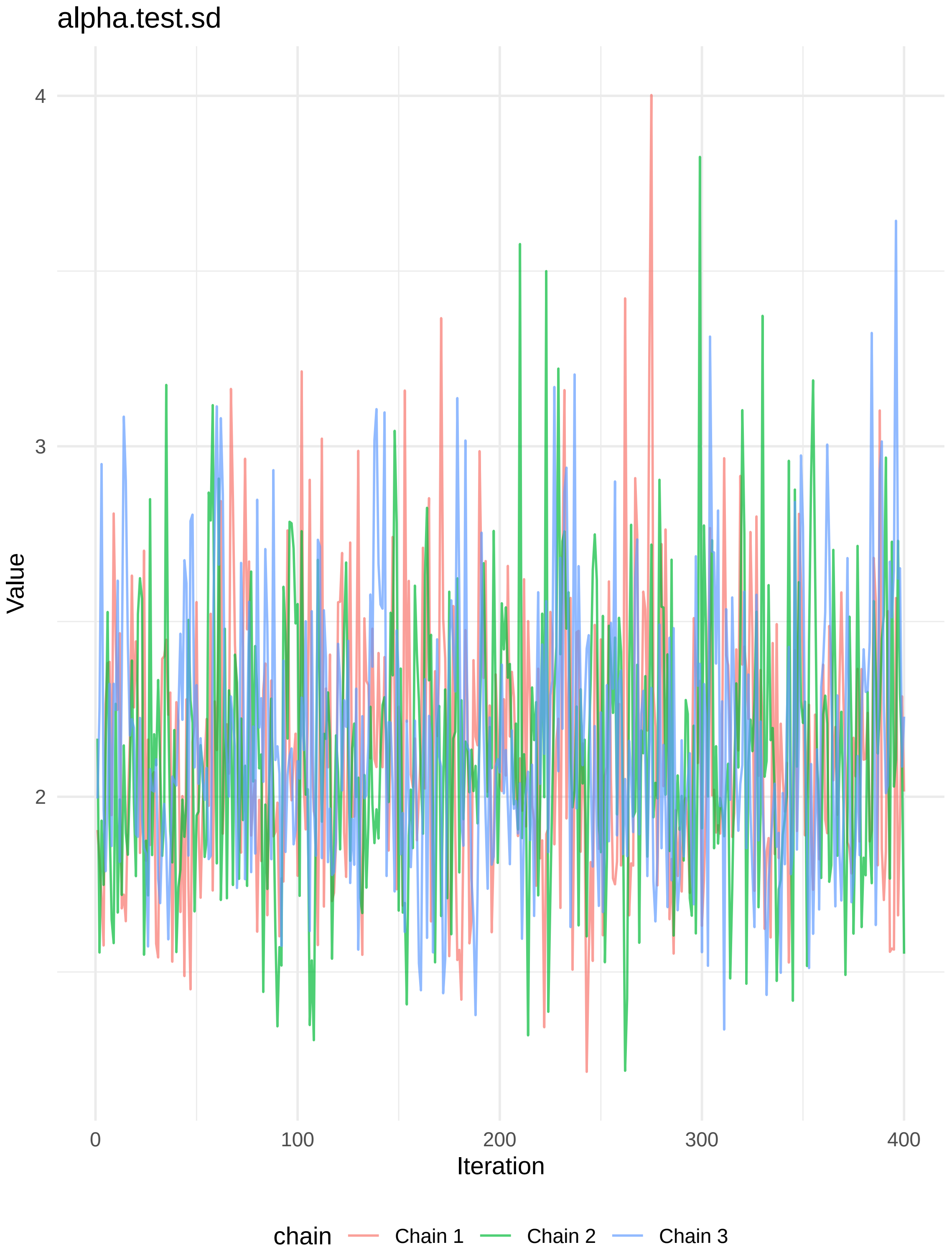

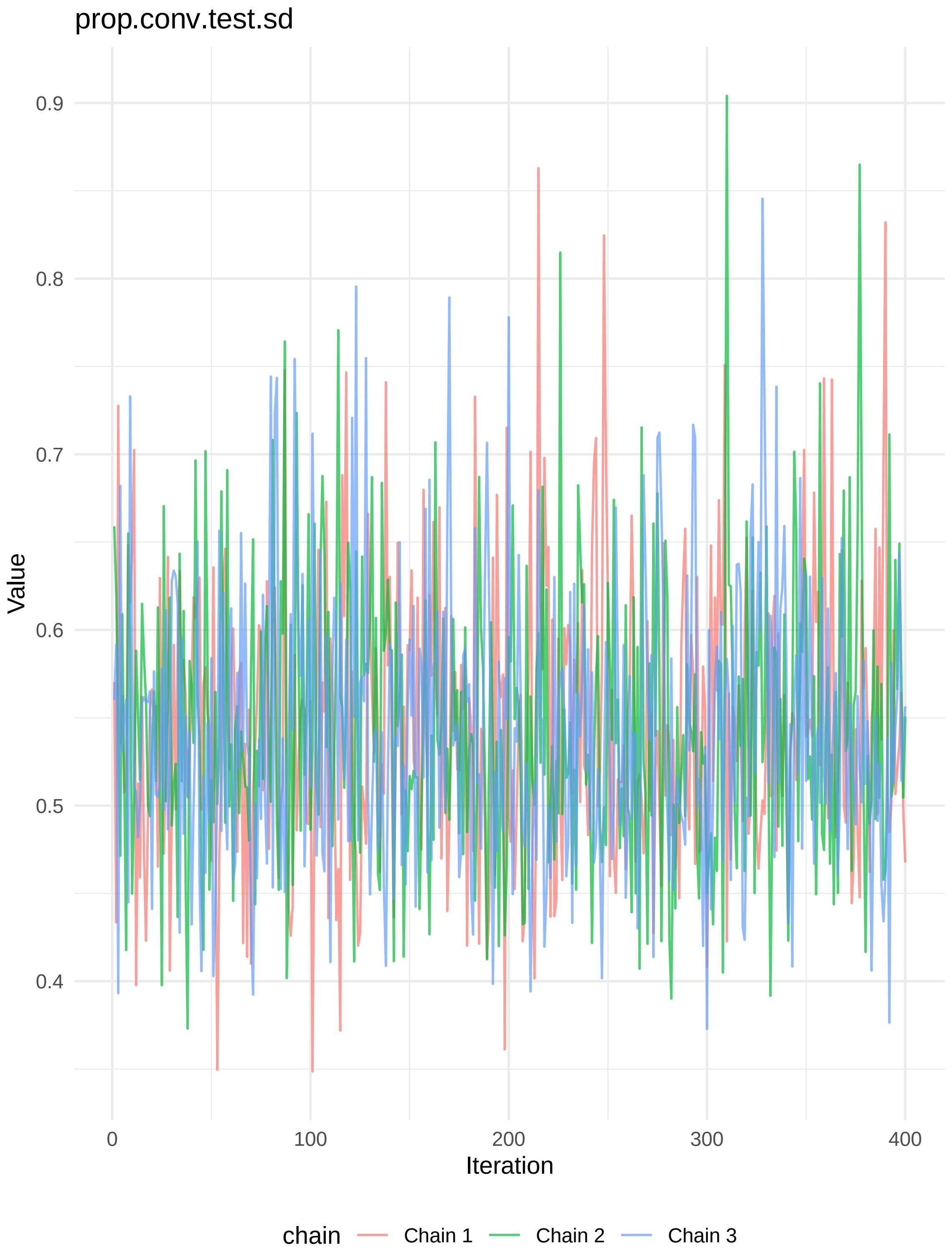

	Point est.	Upper C.I.
shape.conv.intercept	1.005	1.023
scale.conv.intercept	1	1.005
log.lambda1	1.014	1.054
log.lambda2	0.999	0.999
alpha.severity[1]	1.002	1.005
alpha.severity[2]	1.002	1.004
alpha.ag[1]	1.002	1.009
alpha.ag[2]	0.998	0.998
prop.conv.severity[1]	1.002	1.01
prop.conv.severity[2]	1	1.005
prop.conv.severity[3]	1.005	1.012
prop.conv.ag[2]	1.001	1.007
prop.conv.ag[3]	1.006	1.024
alpha.test.sd	1.001	1.007
prop.conv.test.sd	1.001	1.008
