# Supplementary material for: Factors influencing SARS-CoV-2 IgG test sensitivity: A Bayesian analysis of seroconversion and seroreversion by time since infection, test, age and disease severity
Source: PLoS One. 2026 Feb 2;21(2):e0328144. doi: 10.1371/journal.pone.0328144 (PMC12863488; doi:10.1371/journal.pone.0328144)
Supplement: S2 Fig — (PDF) [file pone.0328144.s002.pdf]

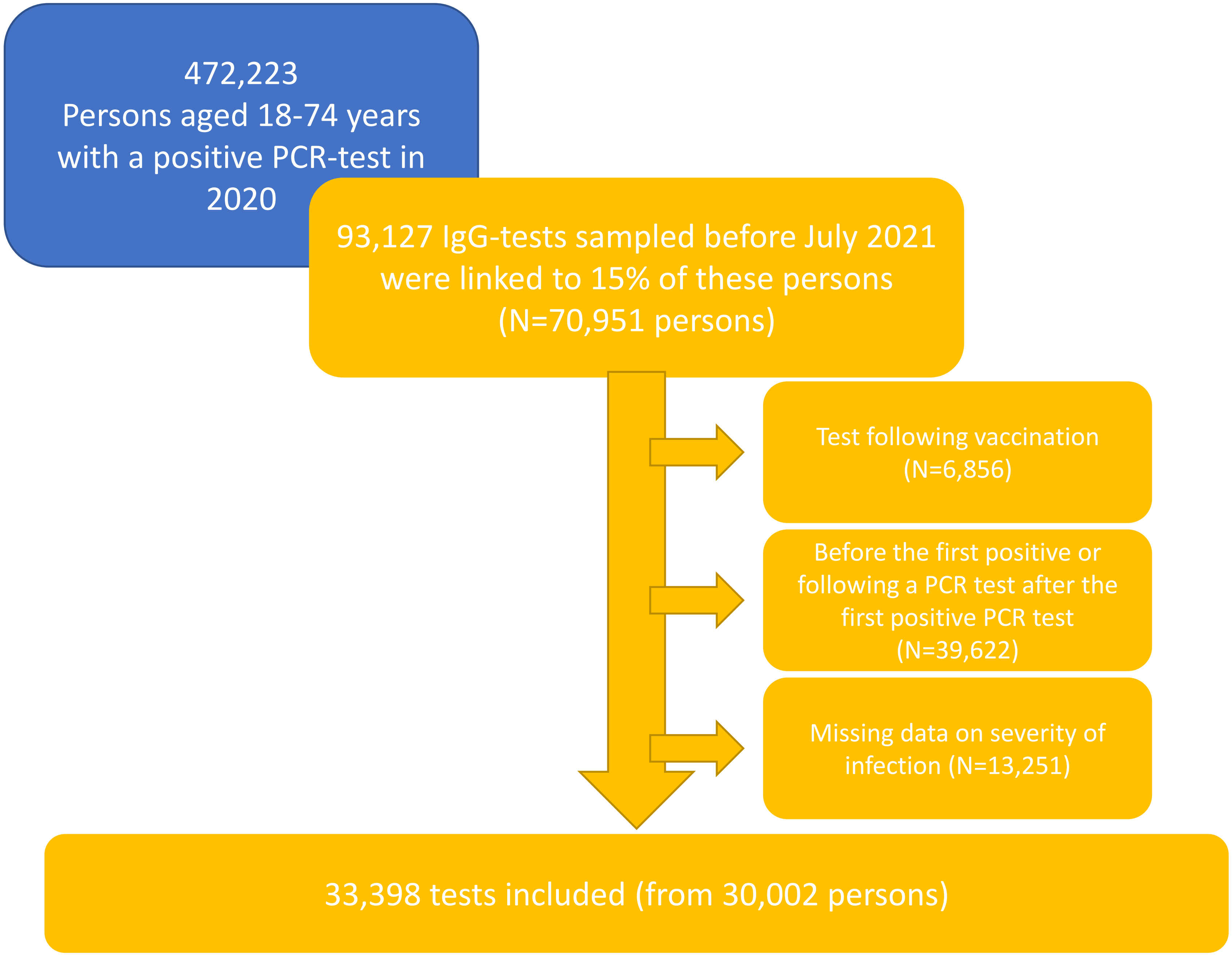
472,223
Persons aged 18-74 years with a positive PCR-test in 2020
93,127 IgG-tests sampled before July 2021 were linked to 15% of these persons (N=70,951 persons)
Test following vaccination (N=6,856)
Before the first positive or following a PCR test after the first positive PCR test (N=39,622)
Missing data on severity of infection (N=13,251)
33,398 tests included (from 30,002 persons)
