# Supplementary figures and images for: Factors influencing SARS-CoV-2 IgG test sensitivity: A Bayesian analysis of seroconversion and seroreversion by time since infection, test, age and disease severity
Source: PLoS One. 2026 Feb 2;21(2):e0328144. doi: 10.1371/journal.pone.0328144 (PMC12863488; doi:10.1371/journal.pone.0328144)

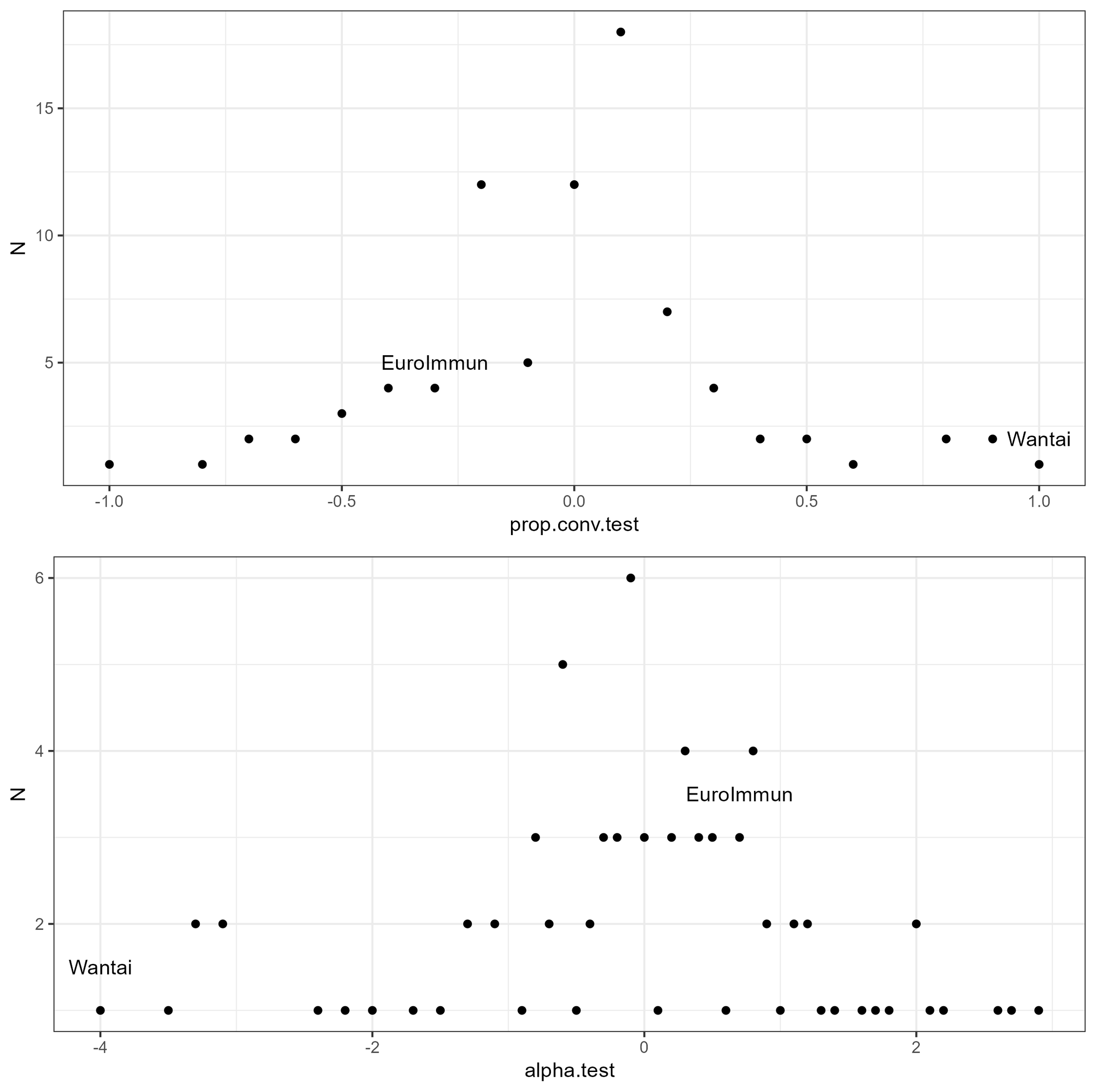

Supplement: S3 Fig — The values associated with the EuroImmun and Wantai tests are annotated. (TIFF) [file pone.0328144.s003.tiff]
