# Supplementary material for: Factors influencing SARS-CoV-2 IgG test sensitivity: A Bayesian analysis of seroconversion and seroreversion by time since infection, test, age and disease severity
Source: PLoS One. 2026 Feb 2;21(2):e0328144. doi: 10.1371/journal.pone.0328144 (PMC12863488; doi:10.1371/journal.pone.0328144)
Supplement: S1 Table — Author refers to the first author, proportion by clinical severity (asymp, symp, hosp) and age group are presented per study. The notes describe the source of these proportions. (DOCX) [file pone.0328144.s004.docx]

S1 Table: Data from published studies on seroconversion after PCR-confirmed infection for the EuroImmun (upper) and Wantai (lower) serological test. Author refers to the first author, proportion by clinical severity (asymp, symp, hosp) and age group are presented per study. The notes describe the source of these proportions.

| Author | prop.asymp | prop.symp | prop.hosp | notes_sev | prop.18-49 | prop.50-64 | prop.65-74 | notes_ag |
| --- | --- | --- | --- | --- | --- | --- | --- | --- |
| Kahre [62] | 0.087 | 0.84 | 0.073 | study | 0.55 | 0.3 | 0.15 | default other (Median 46) |
| Choe [63] | 0 | 1 | 0 | study | 1 | 0 | 0 | study (range 22-40) |
| Alvim [64] | 0 | 0.95 | 0.05 | study (no asymp) | 0.55 | 0.3 | 0.15 | default other |
| Tea [23] | 0 | 0.879518 | 0.120482 | study | 0.5 | 0.4 | 0.1 | study (Median 48 (35-59)) |
| Eberhardt [65] | 0.059 | 0.898 | 0.0433 | study | 0.5 | 0.4 | 0.1 | study (Mean 44 (sd 12.86)) |
| Garritsen [66] | 0.072917 | 0.927083 | 0 | study | 0.45 | 0.35 | 0.2 | default germany (median 50 (IQR 40-59)) |
| Vanshylla [21] | 0.046 | 0.917 | 0.029 | study | 0.55 | 0.3 | 0.15 | study (median 44 (graph of distribution in paper)) |
| Lohse [33] | 0.5 | 0.5 | 0 | study | 0.38 | 0.47 | 0.15 | study (approx supp table 1) |
| Scheiblauer [12] | 0 | 0.84 | 0.16 | study | 0.45 | 0.35 | 0.2 | default germany |

| Author | prop.asymp | prop.symp | prop.hosp | notes_sev | prop.18-49 | prop.50-64 | prop.65-74 | notes_ag |
| --- | --- | --- | --- | --- | --- | --- | --- | --- |
| Cito [67] | 0.5 | 0.45 | 0.05 | default | 0.55 | 0.3 | 0.15 | default other |
| Espenhain [68] | 0.44 | 0.51 | 0.05 | study | 0.64 | 0.26 | 0.1 | default denmark |
| Gudbjartsson [7] | 0.5 | 0.47 | 0.03 | study | 0.64 | 0.26 | 0.1 | default denmark |
| SSI | 0.5 | 0.45 | 0.05 | default | 0.45 | 0.35 | 0.2 | default germany |
| Scheiblauer [12] | 0 | 0.84 | 0.16 | study | 0.45 | 0.35 | 0.2 | default germany |
| Honge [28] | 0.5 | 0.45 | 0.05 | default | 0.765 | 0.207 | 0.028 | study (table 1) |
