# Supplementary material for: Factors influencing SARS-CoV-2 IgG test sensitivity: A Bayesian analysis of seroconversion and seroreversion by time since infection, test, age and disease severity
Source: PLoS One. 2026 Feb 2;21(2):e0328144. doi: 10.1371/journal.pone.0328144 (PMC12863488; doi:10.1371/journal.pone.0328144)
Supplement: S2 Table — The percentage over the complete samples are presented within brackets. (Eg 10.7% of our sample were males, 18–49 years old, 4–11 weeks after symptomatic infection). Belgian laboratory data. (DOCX) [file pone.0328144.s005.docx]

S2 Table: Number of observations included by sex, severity, age group and weeks since positive PCR test. The percentage over the complete samples are presented within brackets. (Eg 10.7% of our sample were males, 18-49 years old, 4-11 weeks after symptomatic infection). Belgian laboratory data.

| Severity | Weeks since positive PCR | Male | | | Female | | | Unk | | |
| --- | --- | --- | --- | --- | --- | --- | --- | --- | --- | --- |
|  |  | [18,50) | [50,65) | [65,75) | [18,50) | [50,65) | [65,75) | [18,50) | [50,65) | [65,75) |
| asymp | [0,4) | 702 (2.1) | 344 (1) | 130 (0.4) | 517 (1.5) | 336 (1) | 151 (0.5) | 28 (0.1) | 7 (0) | 4 (0) |
|  | [4,12) | 1189 (3.6) | 634 (1.9) | 254 (0.8) | 625 (1.9) | 484 (1.4) | 249 (0.7) | 40 (0.1) | 21 (0.1) | 3 (0) |
|  | [12,36) | 1276 (3.8) | 580 (1.7) | 211 (0.6) | 605 (1.8) | 481 (1.4) | 186 (0.6) | 36 (0.1) | 22 (0.1) | 3 (0) |
|  | [36,52) | 91 (0.3) | 28 (0.1) | 7 (0) | 49 (0.1) | 22 (0.1) | 1 (0) | 5 (0) |  |  |
| symp | [0,4) | 924 (2.8) | 510 (1.5) | 210 (0.6) | 488 (1.5) | 447 (1.3) | 208 (0.6) | 18 (0.1) | 8 (0) | 3 (0) |
|  | [4,12) | 3565 (10.7) | 2176 (6.5) | 762 (2.3) | 1464 (4.4) | 1307 (3.9) | 663 (2) | 96 (0.3) | 37 (0.1) | 5 (0) |
|  | [12,36) | 3493 (10.5) | 2088 (6.3) | 630 (1.9) | 1477 (4.4) | 1221 (3.7) | 443 (1.3) | 93 (0.3) | 48 (0.1) | 9 (0) |
|  | [36,52) | 47 (0.1) | 16 (0) | 2 (0) | 15 (0) | 16 (0) | 2 (0) | 2 (0) | 1 (0) |  |
| hosp | [0,4) | 48 (0.1) | 54 (0.2) | 63 (0.2) | 46 (0.1) | 79 (0.2) | 92 (0.3) | 8 (0) | 2 (0) | 7 (0) |
|  | [4,12) | 63 (0.2) | 125 (0.4) | 80 (0.2) | 66 (0.2) | 157 (0.5) | 115 (0.3) |  | 6 (0) | 3 (0) |
|  | [12,36) | 52 (0.2) | 104 (0.3) | 67 (0.2) | 69 (0.2) | 158 (0.5) | 100 (0.3) |  | 4 (0) |  |
|  | [36,52) |  | 1 (0) | 4 (0) | 5 (0) | 3 (0) | 2 (0) |  |  |  |
