# Supplementary material for: Factors influencing SARS-CoV-2 IgG test sensitivity: A Bayesian analysis of seroconversion and seroreversion by time since infection, test, age and disease severity
Source: PLoS One. 2026 Feb 2;21(2):e0328144. doi: 10.1371/journal.pone.0328144 (PMC12863488; doi:10.1371/journal.pone.0328144)
Supplement: S3 Table — (Eg 79.6% of females, 18–49 years old, 12–35 weeks after symptomatic infection had a positive serological test). Belgian laboratory data. (DOCX) [file pone.0328144.s006.docx]

S3 Table: Percentage of seropositivity by sex, severity, age group and weeks since positive PCR test. (Eg 79.6% of females, 18-49 years old, 12-35 weeks after symptomatic infection had a positive serological test). Belgian laboratory data.

| Severity | Weeks since positive PCR | Male | | | Female | | | Unk | | |
| --- | --- | --- | --- | --- | --- | --- | --- | --- | --- | --- |
|  |  | [18,50) | [50,65) | [65,75) | [18,50) | [50,65) | [65,75) | [18,50) | [50,65) | [65,75) |
| asymp | [0,4) | 49.4 | 53.8 | 58.5 | 45.1 | 55.4 | 47.7 | 64.3 | 71.4 | 75.0 |
|  | [4,12) | 75.1 | 76.5 | 79.9 | 75.8 | 77.7 | 80.3 | 92.5 | 95.2 | 66.7 |
|  | [12,36) | 75.1 | 80.3 | 84.8 | 74.4 | 78.8 | 82.8 | 94.4 | 86.4 | 100.0 |
|  | [36,52) | 79.1 | 78.6 | 71.4 | 71.4 | 77.3 | 100.0 | 100.0 |  |  |
| symp | [0,4) | 54.7 | 65.1 | 68.1 | 51.6 | 66.4 | 70.7 | 88.9 | 75.0 | 33.3 |
|  | [4,12) | 81.6 | 88.1 | 89.4 | 84.5 | 89.9 | 91.9 | 95.8 | 97.3 | 100.0 |
|  | [12,36) | 78.1 | 87.3 | 91.4 | 79.6 | 88.5 | 92.6 | 100.0 | 100.0 | 100.0 |
|  | [36,52) | 70.2 | 87.5 | 100.0 | 60.0 | 87.5 | 100.0 | 100.0 | 100.0 |  |
| hosp | [0,4) | 47.9 | 53.7 | 63.5 | 52.2 | 50.6 | 56.5 | 50.0 | 50.0 | 14.3 |
|  | [4,12) | 90.5 | 96.0 | 96.2 | 97.0 | 94.9 | 96.5 |  | 83.3 | 66.7 |
|  | [12,36) | 88.5 | 96.2 | 98.5 | 92.8 | 97.5 | 98.0 |  | 75.0 |  |
|  | [36,52) |  | 100.0 | 100.0 | 100.0 | 100.0 | 100.0 |  |  |  |
