# Supplementary material for: Factors influencing SARS-CoV-2 IgG test sensitivity: A Bayesian analysis of seroconversion and seroreversion by time since infection, test, age and disease severity
Source: PLoS One. 2026 Feb 2;21(2):e0328144. doi: 10.1371/journal.pone.0328144 (PMC12863488; doi:10.1371/journal.pone.0328144)
Supplement: Attachment — Submitted filename: PONE-D-25-34869_review_v0.docx [file pone.0328144.s007.docx]

The submitted paper presents a Bayesian framework for seroconversion and seroreversion analysis including time, age and severity factors, and including both literature and Belgium laboratory data. The methodology proposed appears robust and suggests possible applications in other contexts. The results suggest higher seroconversion for higher ages and as symptoms get more severe, while seroreversion was more common for lower ages and over the light-symptomatic group, almost not occurring over hospitalized patients.

Some studies include gender as a possible variable regarding seroconversion. From your Discussion section: “We opted not to explore sex because initial analysis did not associated sex with large differences in seropositivity after infection… With some claiming a more durable response in females [48].”. Your paper included references to studies showing differences (and also no differences) regarding gender. I believe including this factor in your analysis would make your study even more robust.

Please review your parenthesis usages along the article. In some paragraphs, such as in page 6 lines 116 to 119, there are too many, including parenthesis-inside-parenthesis, which makes the text a bit harder to read. I suggest that you consider changing some descriptions from parenthesis to commas in order to make the text cleaner, for example: “The random variable 𝑆𝑡 (with discrete probability density function (h(𝑆𝑡)) represents…” -> “The random variable 𝑆𝑡, with discrete probability density function h(𝑆𝑡), represents…”

Please review all equations. I suggest that they should all be numbered and referred directly in the text, e.g. “… we include h(𝑆𝑡) as (notation based on Shioda et al. [26]):” -> “… we include h(𝑆𝑡) as shown in Equation X (notation based on Shioda et al. [26]).”

Page 5, line 78. “Whether or not this decrease is antigen-specific is still under debate”. Provide reference regarding this statement.

Page 6, lines 117-119. “To account for **both** the time to seroconversion, the time to seroreversion given seroconversion and the overall proportion that will seroconvert we include h(𝑆𝑡) as (notation based on Shioda et al. [26]):”. In this case, ‘both’ does not fit since h(St) is included to account for three conditions, not two.

Page 6, multiple lines. “$t-(t_{c})$”. Why is $t_{c}$ in parenthesis in this case? Given $t_{c}$ already has the proper underscore, is not a function and no other operation is used inside the parenthesis, could it simply be “$t-t_{c}$” instead? That alternative notation would make the equations and text a bit cleaner by removing the seemingly-unnecessary parenthesis.

Page 9, lines 212-213. “Of these persons 15% (N = 70 951) had IgG tests (N = 93 127) before July 2021.”. As I understand, first number refer to total number of persons with tests, and second number to total number of tests, but that should be clearer in the text what each number is, it is a bit confusing.

Page 13, lines 261-262. “The EuroImmun test was associated with more and faster seroreversion compared to the Wantai test.”. Why? How could the S1/RBD protein antibodies relate to the observed seroreversion differences? Is one test considered of higher quality than the other? The paper’s affirmation could be discussed.

Subsection 4.2.3. This whole section is a small paragraph with two figures. Again, better discussion over the results could be added. What could explain the behaviour of the results obtained by both tests being so different? How these align (or deviate from) with the laboratory results? How these relate to results obtained by other papers in the literature? Among other discussions.

Fig 3, page 14, line 279. “… scaled Weibull-Wi-exponential distribution…”. There is a small typo in the figure label. Please do a double-check over the entire paper for small typos like this.

Discussion section, pages 15-16. Second paragraph is enormous. Please break it down in multiple smaller paragraphs.

All figures. Figures look of low quality. Please increase the resolution/quality/DPI of the figures.
