# Supplementary material for: Factors influencing SARS-CoV-2 IgG test sensitivity: A Bayesian analysis of seroconversion and seroreversion by time since infection, test, age and disease severity
Source: PLoS One. 2026 Feb 2;21(2):e0328144. doi: 10.1371/journal.pone.0328144 (PMC12863488; doi:10.1371/journal.pone.0328144)
Supplement: Attachment — Submitted filename: Review of Manuscript PONE-DS-25-34869.docx [file pone.0328144.s008.docx]

**Review of Manuscript PONE-D-25-34869**

The objectives of this study were to quantify SARS-CoV-2 seroconversion and reversion by time since PCR-confirmed infection, age and disease severity using data from the national/centralized database of Belgium as well as from various Laboratory based SARS-CoV-2 IgG antibody data.

The findings of this study are not novel or unique, but with the use of new approach, the findings were confirmed.

Specific comments are given below:

**Laboratory data**

1. It is not clear to me how the Government database or registries on PCR test results was linked with SARS-CoV-2 IgG test results done in various laboratories? Please explain/describe how the government PCR data IDs can be matched with participant IDs for antibody response with a pseudo-identifier. There is a description in lines 158 to 162, but the description is not adequate.

A section is needed to describe the LINK-VACC project, which appears to be a European registry for COVID-19 patients or vaccination. Please state what information are available from the database, e.g. age, sex, PCR testing, vaccination history etc. Whether SARS-CoV-2 IgG titers are available in database or not, how the pseudo-identifier were created and linked with individual antibody titers. This section is essential for the readers to understand and follow the design and approaches of the paper.

2. Can you please insert information about when (time line) mass vaccination started in Belgium?
3. It seems that data were sometimes were used from other European studies, not clear why this was done and whether it is justified to use these data to support the Belgian study.

**Methods**

- The model description is mathematically detailed — the readers may not be expected to follow the equations; simplified narrative first (with equations in supplementary materials) may improve readability and ease of understanding.
- The authors used a hierarchical Bayesian model — please provide a brief explanation about *why Bayesian inference* was chosen over classical alternatives (e.g., was it for better handling of uncertainty, incorporation of prior information)?
- Please give a rationale as to why “non-informative priors (normal with SD=100)” — was a reasonable choice, and whether sensitivity analyses were done with alternative priors?
- MCMC: Were convergence diagnostics besides Gelman-Rubin (e.g., trace plots, ESS) used and do they confirm robustness?

**Results**

- Numbers included: Could a flow diagram (e.g., exclusions step-by-step) help clarify the selection process of samples?
- Seroconversion and seroreversion: Figures are referenced, but the text could highlight *key takeaways* more explicitly (e.g., “By week 5, >95% had seroconverted, but durability varied strongly by test and severity”).
- Hierarchical model: Author note “effect of test/laboratories in supporting information” — summarizing the magnitude of variation in the main text may strengthen the meaning.
- Goodness of fit: Did the author provide any quantitative fit statistics in addition to plots (e.g., WAIC, DIC)?
