# Supplementary material for: Factors influencing SARS-CoV-2 IgG test sensitivity: A Bayesian analysis of seroconversion and seroreversion by time since infection, test, age and disease severity
Source: PLoS One. 2026 Feb 2;21(2):e0328144. doi: 10.1371/journal.pone.0328144 (PMC12863488; doi:10.1371/journal.pone.0328144)
Supplement: Attachment — Submitted filename: Response to Reviewers.docx [file pone.0328144.s009.docx]

**Review of Manuscript PONE-D-25-34869**

The objectives of this study were to quantify SARS-CoV-2 seroconversion and reversion by time since PCR-confirmed infection, age and disease severity using data from the national/centralized database of Belgium as well as from various Laboratory based SARS-CoV-2 IgG antibody data.

The findings of this study are not novel or unique, but with the use of new approach, the findings were confirmed.

We would like to thank the reviewer their comments. Their contribution aided in improving the paper. We would like to start by mentioning that during our sensitivity analyses (requested by the reviewer), we identified an opportunity to improve model convergence. We revised the model specification by removing intercepts from the linear predictors for α (mixture parameter) and proportion converted, and adjusted the reference levels accordingly. This specification improves parameter identifiability, particularly for α, by anchoring the mixture parameter near zero for the group showing slowest reversion (elderly, hospitalized patients).

**Importantly, all substantive results remain unchanged.** The revised model specification affects only the technical parameterization, not the underlying biological interpretations or conclusions. The results are robust to this model change.

Specific comments are given below:

**Laboratory data**

1. It is not clear to me how the Government database or registries on PCR test results was linked with SARS-CoV-2 IgG test results done in various laboratories? Please explain/describe how the government PCR data IDs can be matched with participant IDs for antibody response with a pseudo-identifier. There is a description in lines 158 to 162, but the description is not adequate.

A section is needed to describe the LINK-VACC project, which appears to be a European registry for COVID-19 patients or vaccination. Please state what information are available from the database, e.g. age, sex, PCR testing, vaccination history etc. Whether SARS-CoV-2 IgG titers are available in database or not, how the pseudo-identifier were created and linked with individual antibody titers. This section is essential for the readers to understand and follow the design and approaches of the paper.

We reordered the method section and included more information on the LINKVACCC-project.

(line 106) Reporting of laboratory results to a centralized database was mandatory during the COVID-19 pandemic in Belgium. While SARS-CoV-2 IgG testing was not required in any situation, a large number of IgG tests were performed and reported to the centralized database. This database could be linked to other databases. For this study, linkage was made with the contact tracing database (results from case interviews, including self-reported symptoms), the vaccinnet+ database (COVID-19 vaccination registry), the DEMOBEL database (age, sex, place of residence) and the clinical hospital surveillance. Linkage was based on a pseudonymized identifier obtained from the encryption of an individual’s national registry number and performed in LINK-VACC, a secure pseudonymized environment hosted by Healthdata.be at the Belgian Institute for Public Health.

2. Can you please insert information about when (time line) mass vaccination started in Belgium?

We added the following lines to the methods section.

(Line 135) Belgium started the mass roll-out of the vaccination campaign in January 2021, prioritizing nursing home residents and healthcare workers. The further roll-out was age-based, with persons below the age of 65 years invited from April 2021 onwards. For individuals in our study cohort who subsequently received COVID-19 vaccination, we excluded any IgG test results obtained after their vaccination date.

3. It seems that data were sometimes were used from other European studies, not clear why this was done and whether it is justified to use these data to support the Belgian study.

Our study aimed to include all relevant information on the Wantai and EuroImmun test. The Belgian data in combination with published longitudinal datasets allowed for a significant sample size, which in turn allowed us to estimate the effects of age and disease severity. We tried to include this rationale more clearly in the paper

(Line 99, start methods section) Data on cases was collected from Belgian laboratories and published studies. Belgian laboratory data contributed large sample sizes, but relied on reporting laboratory as a proxy for test type. Published longitudinal studies provided small, well-characterized cohorts with explicit test identification. Our approach is analogous to meta-analytic methods that synthesize evidence across data sources to include all information and increase statistical precision.

The abstract has also been slightly rephrased to include this rationale.

**Methods**

- The model description is mathematically detailed — the readers may not be expected to follow the equations; simplified narrative first (with equations in supplementary materials) may improve readability and ease of understanding.

We rewrote the methods section. While we would suggest to continue including some of the formulas in the main text, we have improved and expanded the non-mathematical description of the model in the methods.

(line 167) We modeled two immunological processes: seroconversion and seroreversion. Seroconversion is the process of reaching a detectable level of antibodies after infection. Seroreversion is the process of losing that detectable level since having first attained it. These processes are represented by two time-to-event distributions: a Weibull distribution for time to seroconversion ($T_{c}$) and a bi-exponential distribution for time to seroreversion after conversion ($T_{r}$). The Weibull distribution was selected because it can flexibly model time-to-event data. The bi-exponential distribution allowed us to include a mixture of a fast and slow decrease. By combining these distributions, we calculate the proportion of individuals who remain seropositive at any given time after infection ($S_{t}$), accounting for age, disease severity, and test type.

- The authors used a hierarchical Bayesian model — please provide a brief explanation about *why Bayesian inference* was chosen over classical alternatives (e.g., was it for better handling of uncertainty, incorporation of prior information)?

We chose a hierarchical Bayesian framework for several reasons. First, the hierarchical structure naturally accommodates the multi-level nature of our data, where individual test results are nested within laboratories/tests, which in turn share common underlying antibody kinetics. We could estimate both global parameters and group-specific parameters within the same model with proper propagation of uncertainty across all levels. Second, our model includes complex non-linear functions (Weibull and bi-exponential distributions) with constraints (e.g., probabilities must be between 0 and 1), making likelihood-based inference challenging.

(Line 204) We opted for Bayesian inference since it allows for simultaneous estimation of global parameters (shape of seroconversion and seroreversion curves), group-specific parameters (effects of age and severity), and test/laboratory-specific random effects, with appropriate propagation of uncertainty across all levels. In addition, our model includes complex non-linear function (Weibull and bi-exponential distributions), which would make likelihood-based inference challenging. We evaluated multiple approaches for modelling antibody reversion kinetics, including linear decay functions and various time-to-event distributions such as Weibull. After comparative analysis, we selected a bi-exponential distribution. This model optimally captured the biphasic nature of antibody dynamics: an initial rapid decline followed by a slower, plateau-like phase. While $\alpha_{r}$ was allowed to vary by severity, age group, and test, we determined through model selection that the decay rate parameters $\lambda_{r.fast}$ and $\lambda_{r.slow}$ could be treated as global parameters without significant loss of fit. Multiple model structures were evaluated using Bayesian Information Criterion (BIC) and convergence diagnostics, including interaction terms. All credible intervals (CrI) reported are 95% intervals unless otherwise stated.

- Please give a rationale as to why “non-informative priors (normal with SD=100)” — was a reasonable choice, and whether sensitivity analyses were done with alternative priors?

Yes we did sensitivity analyses with different priors. A diffuse normal prior (0, SD=100) puts quite a lot of weight around 0 and 1 on the logit scale. We performed sensitivity analysis with SD=5. Results were not sensitive to these choices. This is unsurprising given the large number of observations included.

(Line 218) We used diffuse normal priors (mean = 0, SD = 100) for model coefficients. Sensitivity analyses with more informative priors (SD = 5) produced nearly identical parameter estimates.

- MCMC: Were convergence diagnostics besides Gelman-Rubin (e.g., trace plots, ESS) used and do they confirm robustness?

We included trace plots in the supplementary materials to illustrate convergence. We also explicitly included the output of the Gelman-Rubin statistic (S1 Fig).

**Results**

- Numbers included: Could a flow diagram (e.g., exclusions step-by-step) help clarify the selection process of samples?

We included a flow diagram in the supplementary materials (S2 Fig).

- Seroconversion and seroreversion: Figures are referenced, but the text could highlight *key takeaways* more explicitly (e.g., “By week 5, >95% had seroconverted, but durability varied strongly by test and severity”).

We now include several such instances:

(line 263) For context, among symptomatic individuals aged 18-49 years, the predicted seroconversion at week 6 ranged from 72% to 95% depending on the test, while the proportion remaining seropositive at week 50 ranged from 32% to 95%.

(line 287) 50 Weeks after a symptomatic infection only 64% 18-49 year-olds, remained seropositive with EuroImmun compared to 95% with Wantai. Differences were smaller after hospitalization and for older age groups. For example, in 65-74 year-olds after hospitalization 96% remained seropositive with EuroImmun compared to 98% with Wantai.

- Hierarchical model: Author note “effect of test/laboratories in supporting information” — summarizing the magnitude of variation in the main text may strengthen the meaning.

We expanded on those results:

(line 260) Substantial variation in antibody dynamics was attributed to test/laboratory. The standard deviation of test-specific random effects for the mixture parameter $\alpha_{r}$ was 2.15 (CrI: 1.52-3.02). For proportion seroconverting, the SD was 0.55 (95% CrI: 0.41-0.72), corresponding to OR = 0.34 to OR = 2.98 across tests.

- Goodness of fit: Did the author provide any quantitative fit statistics in addition to plots (e.g., WAIC, DIC)?

We assess model fit through posterior predictive checks (Figures 3 and ), which demonstrate that our model captures the key patterns in the data. During model development, we compared alternative specifications using BIC, which led to our selection of the Weibull-bi-exponential structure.

We appreciate the reviewer's interest in quantitative fit measures. While we could report WAIC or DIC, these metrics are most informative when comparing multiple candidate models. Given that we present a single final model, and that our posterior predictive checks show good agreement between observed and predicted values, we felt additional information criteria would subtract focus from the main model’s results.

The submitted paper presents a Bayesian framework for seroconversion and seroreversion analysis including time, age and severity factors, and including both literature and Belgium laboratory data. The methodology proposed appears robust and suggests possible applications in other contexts. The results suggest higher seroconversion for higher ages and as symptoms get more severe, while seroreversion was more common for lower ages and over the light-symptomatic group, almost not occurring over hospitalized patients.

We would like to thank the review for the comments. We tried to improve our work accordingly. We would like to start by mentioning that during our sensitivity analyses (requested by the reviewer), we identified an opportunity to improve model convergence. We revised the model specification by removing intercepts from the linear predictors for α (mixture parameter) and proportion converted, and adjusted the reference levels accordingly. This specification improves parameter identifiability, particularly for α, by anchoring the mixture parameter near zero for the group showing slowest reversion (elderly, hospitalized patients).

**Importantly, all substantive results remain unchanged.** The revised model specification affects only the technical parameterization, not the underlying biological interpretations or conclusions. The results are robust to this model change.

Some studies include gender as a possible variable regarding seroconversion. From your Discussion section: “We opted not to explore sex because initial analysis did not associated sex with large differences in seropositivity after infection… With some claiming a more durable response in females [48].”. Your paper included references to studies showing differences (and also no differences) regarding gender. I believe including this factor in your analysis would make your study even more robust.

During initial analysis on the Belgian data, we did include sex-main effects in the analysis for both the proportion seroconverted and the mixture of fast and slow decay, but none were significant. To truly have this as a study results, however, we feel that we should treat it like age and severity. Which would mean also exploring sex from the data found in published longitudinal studies. Unfortunately this information was missing from many of these studies.

We included additional tables in the supplementary materials that present the number of samples and test positivity by sex (supplementary tables 2 and 3). From the tables it can be seen that sex-differences in test positivity by age, severity and time since infection are minimal. We now more explicitly refer to the initial analysis by including this information in both the methods and discussion section.

(Line 125, methods) We explored sex as an additional covariate, but initial analysis did not indicate a significant association between sex and test positivity. Descriptive statistics for age, severity and sex are included in tables S2 and S3.

(line 386, discussion, limitations) We did not fully explore sex as a cofactor as this information was missing in many of the included longitudinal studies. In our initial analysis, limited to the Belgian data, we found no evidence of heterogeneity in antibody persistence by sex. Some studies have come to a similar conclusion [61], while others claimed a more durable response in females [48].

Please review your parenthesis usages along the article. In some paragraphs, such as in page 6 lines 116 to 119, there are too many, including parenthesis-inside-parenthesis, which makes the text a bit harder to read. I suggest that you consider changing some descriptions from parenthesis to commas in order to make the text cleaner, for example: “The random variable 𝑆𝑡 (with discrete probability density function (h(𝑆𝑡)) represents…” -> “The random variable 𝑆𝑡, with discrete probability density function h(𝑆𝑡), represents…”

We have made several such improvements throughout the manuscript and reworked the methods section. Focussing specifically on the use of parenthesis, but also on less clear or redundant notation in general.

**(Line 176)** In mathematical terms: the random variable $S_{t}$, with discrete probability density function $h(S_{t})$, represents the proportion of seropositive cases at week $t$ after infection…

Please review all equations. I suggest that they should all be numbered and referred directly in the text, e.g. “… we include h(𝑆𝑡) as (notation based on Shioda et al. [26]):” -> “… we include h(𝑆𝑡) as shown in Equation X (notation based on Shioda et al. [26]).”

We have reworked the method section. We have tried to make it more readable by remove redundant notation and repeated information in the text. As per other reviewer request, we expanded the non-mathematical description of the method and made a clearer distinction with the more mathematical part. We have numbered the equations and referred to them in the text.

Page 5, line 78. “Whether or not this decrease is antigen-specific is still under debate”. Provide reference regarding this statement.

We have reformulated this sentence to indicate that there is indeed quite some evidence that anti-N decreases faster than anti-S, but also that it is difficult to give any definitive statement as the difference in antibodies is accompanied by a difference in assays and other factors, like vaccination, might also play a role.

(Line 78) This decrease shows antigen-specific patterns. Antibodies associated with S1 are more durable than those associated with the N-protein [2,11–16] , though the magnitude of this difference may vary by assay type and vaccination status [17].

Page 6, lines 117-119. “To account for **both** the time to seroconversion, the time to seroreversion given seroconversion and the overall proportion that will seroconvert we include h(𝑆𝑡) as (notation based on Shioda et al. [26]):”. In this case, ‘both’ does not fit since h(St) is included to account for three conditions, not two.

Agreed, we have removed the ‘both’

Page 6, multiple lines. “$t-(t_{c})$”. Why is $t_{c}$ in parenthesis in this case? Given $t_{c}$ already has the proper underscore, is not a function and no other operation is used inside the parenthesis, could it simply be “$t-t_{c}$” instead? That alternative notation would make the equations and text a bit cleaner by removing the seemingly-unnecessary parenthesis.

Indeed, the parentheses are redundant. We, mistakenly, felt that they might improve the visual clarity, but we agree to remove them. As previously mentioned we reworked the methods section to specifically reduce what could be considered unnecessary.

Page 9, lines 212-213. “Of these persons 15% (N = 70 951) had IgG tests (N = 93 127) before July 2021.”. As I understand, first number refer to total number of persons with tests, and second number to total number of tests, but that should be clearer in the text what each number is, it is a bit confusing.

Your understanding is correct. We reformulated this sentence to improve its flow and make it clearer.

(Line 228) Of these persons 15% (N = 70,951) had IgG tests before July 2021. The total number of IgG tests associated with these persons was 93,127.

Page 13, lines 261-262. “The EuroImmun test was associated with more and faster seroreversion compared to the Wantai test.”. Why? How could the S1/RBD protein antibodies relate to the observed seroreversion differences? Is one test considered of higher quality than the other? The paper’s affirmation could be discussed.

Subsection 4.2.3. This whole section is a small paragraph with two figures. Again, better discussion over the results could be added. What could explain the behaviour of the results obtained by both tests being so different? How these align (or deviate from) with the laboratory results? How these relate to results obtained by other papers in the literature? Among other discussions.

We included an additional reference and some discussion. We have to admit that, while interesting, this is outside the scope of this study. We do make the observation, but we cannot point to specific reasons for why higher reversion is observed with EuroImmun as compared to Wantai. The value of our study lies in quantifying the observation.

(Line 353) Our study included two commonly used assays: EuroImmun and Wantai. EuroImmun detects antibodies to the S1 domain while Wantai targets the receptor-binding domain (RBD). In addition to differences in the targeted antigen, assay-specific characteristics - including which antibody classes are detected (total antibodies vs. IgG only), manufacturer cut-off thresholds, and epitope-specific binding characteristics - are expected to result in different time-varying sensitivity [17].

We did also expand on the observed differences between tests.

(Line 286) The EuroImmun test was associated with more and faster seroreversion compared to the Wantai test. 50 Weeks after a symptomatic infection only 64% 18-49 year-olds, remained seropositive with EuroImmun compared to 95% with Wantai. Differences were smaller after hospitalization and for older age groups. For example, in 65-74 year-olds after hospitalization 96% remained seropositive with EuroImmun compared to 98% with Wantai.

Our results agree with previously published results. This is perhaps unsurprising as our model is partly fitted to these data (in addition to other data sources). Other meta-analyses have also observed seropositivity to be test-specific (eg Owusu-Boaitey et al.).

Discussion of previously published estimates for EuroImmun and Wantai:

(line 331) For example, for the EuroImmun serological test sensitivity estimates have been reported ranging from 94% to 53%. The manufacturer reported a sensitivity ≥10 days post symptom onset of 94.4%. Researchers in South Africa estimated sensitivity at 64.1% [6], Public Health England at 72%, 74.5% [35], 77.2% [36] and a study on Antarctic cruise passengers at 81% declining to 76% at 3 months and 53% at 1 year [37].

(line 362) Previous research reported 94.2%-98% seropositivity without seroreversion for at least 13-15 months after infection for Wantai [2,9,23,28,38].

Fig 3, page 14, line 279. “… scaled Weibull-Wi-exponential distribution…”. There is a small typo in the figure label. Please do a double-check over the entire paper for small typos like this.

Indeed, thank you for spotting this typo. We double-checked the paper.

Discussion section, pages 15-16. Second paragraph is enormous. Please break it down in multiple smaller paragraphs.

We rewrote parts of the discussion and broke the paragraph into smaller paragraphs.

There are now separate parts more focused on the differences observed between Wantai and EuroImmun.

All figures. Figures look of low quality. Please increase the resolution/quality/DPI of the figures.

This might be due to how the journal converted our images, we will see if the quality of the images can be maintained after uploading them. We do provide them in 300 dpi.
