# Supplementary material for: Factors influencing SARS-CoV-2 IgG test sensitivity: A Bayesian analysis of seroconversion and seroreversion by time since infection, test, age and disease severity
Source: PLoS One. 2026 Feb 2;21(2):e0328144. doi: 10.1371/journal.pone.0328144 (PMC12863488; doi:10.1371/journal.pone.0328144)
Supplement: Attachment — Submitted filename: ResponseToReviewers2.docx [file pone.0328144.s010.docx]

Reviewer #1:

Additional review of some equations required - some parenthesis are bigger than others, for example in equation 2. Also, please set the equation number to be right-aligned for all equations, equation (1) for example is not right aligned

Thank you for spotting these formatting issues. We updated the equations.

Reviewer #3:

Thank you for taking the time again to go through this paper and improve our work.

Abstract

Suggestions for improvement: Clarity: Some sentences are long and dense. Breaking them into shorter sentences will improve readability.

Throughout the text there are several instances where we tried to decrease both the complexity and length of the sentences. Examples include the ‘objectives’ (end of introduction) and methods (abstract) section, but there were several changes throughout the paper:

Before:

"We used a hierarchical Bayesian model to estimate distributional parameters of a scaled Weibull-bi-exponential distribution for the time-varying sensitivity of qualitative serological test results obtained after PCR-confirmed SARS-CoV-2 infection."

After:

(Line 29) "We used a hierarchical Bayesian model to estimate time-varying sensitivity of serological tests following PCR-confirmed infection. The model employed a scaled Weibull-bi-exponential distribution."

Consistency of terminology: Ensure uniform use of “seroconversion” and “seroreversion” throughout.

We reviewed the document and changed instances of ‘reversion‘/’conversion’ to ‘seroreversion’/’seroconversion’.

Results section in abstract: Consider highlighting the main takeaway with a short sentence summarizing seropositivity durability by test type and severity.

We slightly rewrote the abstract to include both takeaways more clearly and to include public health implications.

(line 45) These findings highlight the need for test-specific, time-varying sensitivity adjustments in seroprevalence studies. Such adjustments are crucial for translating seroprevalence results to cumulative incidence estimates.

Objectives

The objectives paragraph is slightly long and technical.

We rewrote this part.

(line 92) We aimed to estimate time-varying, test-specific sensitivity in relation to time since infection. We also quantified the effects of age and clinical severity on this sensitivity. To estimate sensitivity, we included all data: data from Belgian laboratories and international data from the literature.

Methods

Consider simplifying: Methods / Model Structure

Suggestions: Complexity / readability: The methods are very technical. Consider providing a simpler overview first for readers less familiar with Bayesian modeling.

Keep the equations, but a brief intuitive explanation in words could help

With the paragraph directly following the ‘model structure’ heading, we aim to introduce the concepts, introducing technical/mathematical jargon, but avoiding equations/too technical parts.

(Line 167) We modeled two immunological processes: seroconversion and seroreversion. Seroconversion is the process of reaching a detectable level of antibodies after infection. Seroreversion is the process of losing that detectable level after having first attained it. We represented these as time-to-event distributions: a Weibull distribution for time to seroconversion ($T_{c}$) and a bi-exponential distribution for time to seroreversion ($T_{r}$). The Weibull distribution flexibly accommodates varying seroconversion rates. The bi-exponential distribution captures the biphasic nature of antibody decay—an initial rapid decline followed by slower waning. By combining these distributions, we calculated the proportion of individuals remaining seropositive at any given time since infection ($S_{t}$), with parameters varying by age, disease severity, and test type. We used Bayesian inference to estimate model parameters from observed test results, allowing us to quantify uncertainty in seroconversion and seroreversion estimates.

Flow: Subheadings like “Data sources,” “Exclusion criteria,” “Bayesian model,” and “Sensitivity analysis” could improve readability.

We added subheadings to the methods-section.

Results

Consider summarizing key points at the beginning of the results section for readers to get the main takeaway quickly.

We presented our key findings at the start of the discussion:
(Line 326) Our key findings demonstrate that seropositivity over time since SARS-CoV-2 infection is significantly influenced by three factors: the specific serological test used, the age of the individual, and the severity of the initial infection.

Provide clear interpretation of CrIs and ORs in plain language for broader readership.

We introduced additional sentences:

(Line 201) The Odds Ratios (OR) obtained from these coefficients quantify the relative odds of seroconversion (equation 3) or fast decay (equation 4) between groups.

(Line 230) All credible intervals (CrI, the Bayesian analogue of confidence intervals) are 95% unless otherwise stated.

Throughout the text there are several instances where we state relationships explicitly. For example:

(Line 260) Higher seroconversion was associated with older age: for 50-64 year-olds the OR was 1.29 (CrI: 1.13-1.45) and for 65-74 year-olds the OR was 1.65 (CrI: 1.41-1.97) compared to the youngest age group (18-49 years old) (Table 2).

Conclusion

Could briefly mention implications for public health, e.g., improving seroprevalence studies or guiding selection of serological tests.

We rewrote the conclusion to explicitly include these implications:

(Line 398) In conclusion, our study provided a comprehensive framework for estimating time-varying, test-specific sensitivity in serological testing for SARS-CoV-2, accounting for the critical factors of disease severity, age, and time since infection. The results demonstrate that older age (50-74 years) and more severe infection lead to higher and more durable seropositivity. Our findings have several practical implications. First, test selection substantially affects sensitivity, particularly at longer intervals post-infection; the Wantai test has higher initial sensitivity which is maintained while EuroImmun showed considerable waning of its already lower sensitivity. Second, seroprevalence estimates from populations differing in age or severity composition are not directly comparable without adjustment. Third, sensitivity estimates derived from hospitalized cohorts—common in validation studies—will overestimate sensitivity in general populations with milder infections.
